# Supplementary figures and images for: Endothelial lipase mediates efficient lipolysis of triglyceride-rich lipoproteins
Source: PLoS Genet. 2021 Sep 20;17(9):e1009802. doi: 10.1371/journal.pgen.1009802 (PMC8483387; doi:10.1371/journal.pgen.1009802)

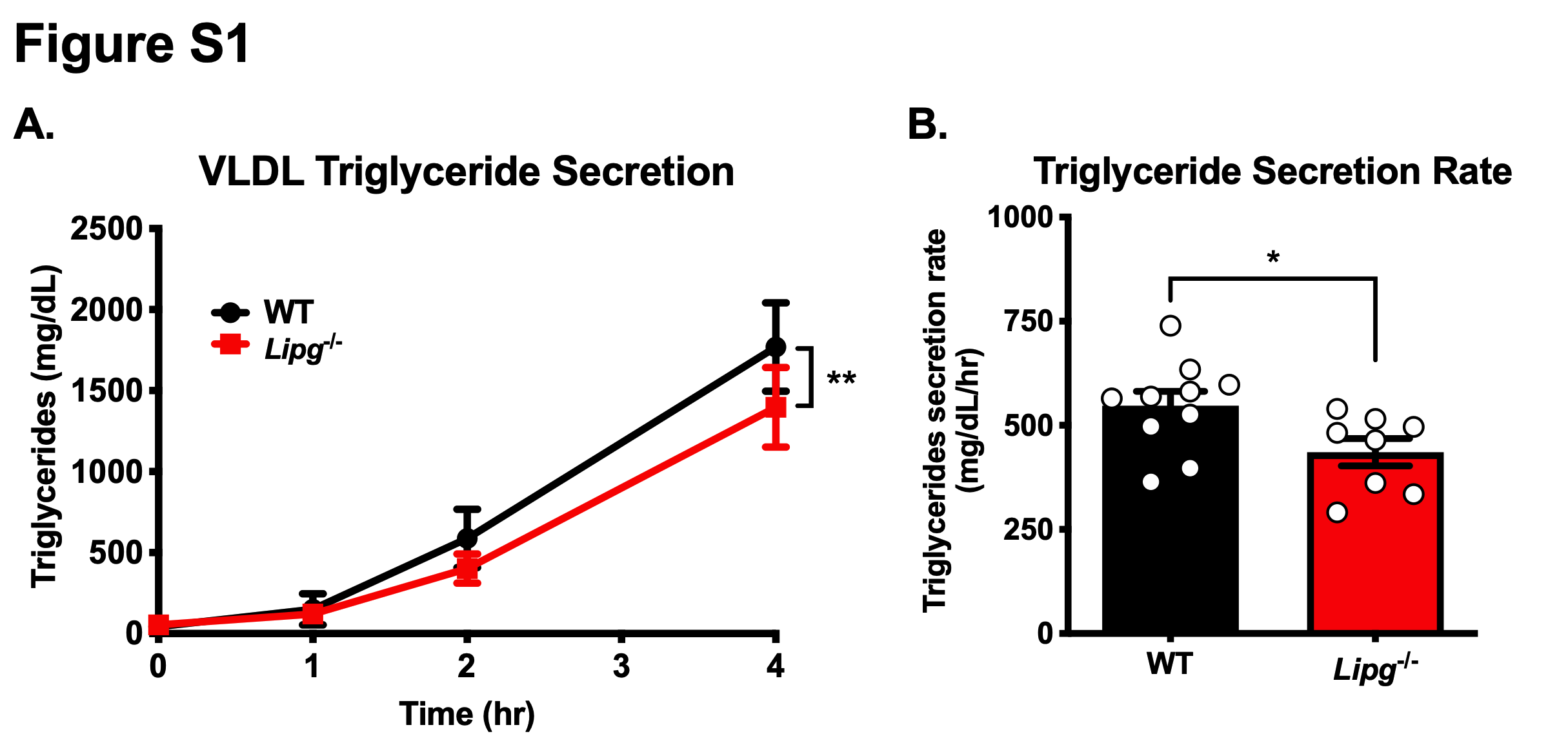

Supplement: S1 Fig — A. Plasma triglycerides in mice at the indicated timepoints following intraperitoneal injection of poloxamer P407, a competitive inhibitor of LPL activity. Plasma TGs were measured by colorimetric biochemical assays. B. VLDL-TG secretion rates calculated from the slope of the curves from (A) using the 1, 2, and 4 hour timepoints. **P<0.01, repeated factor 2-way ANOVA comparing WT and Lipg-/- groups (A). *P<0.05, student’s unpaired T-test comparing WT and Lipg-/- groups (B). Data is expressed as mean ± S.E.M. (TIF) [file pgen.1009802.s001.tif]

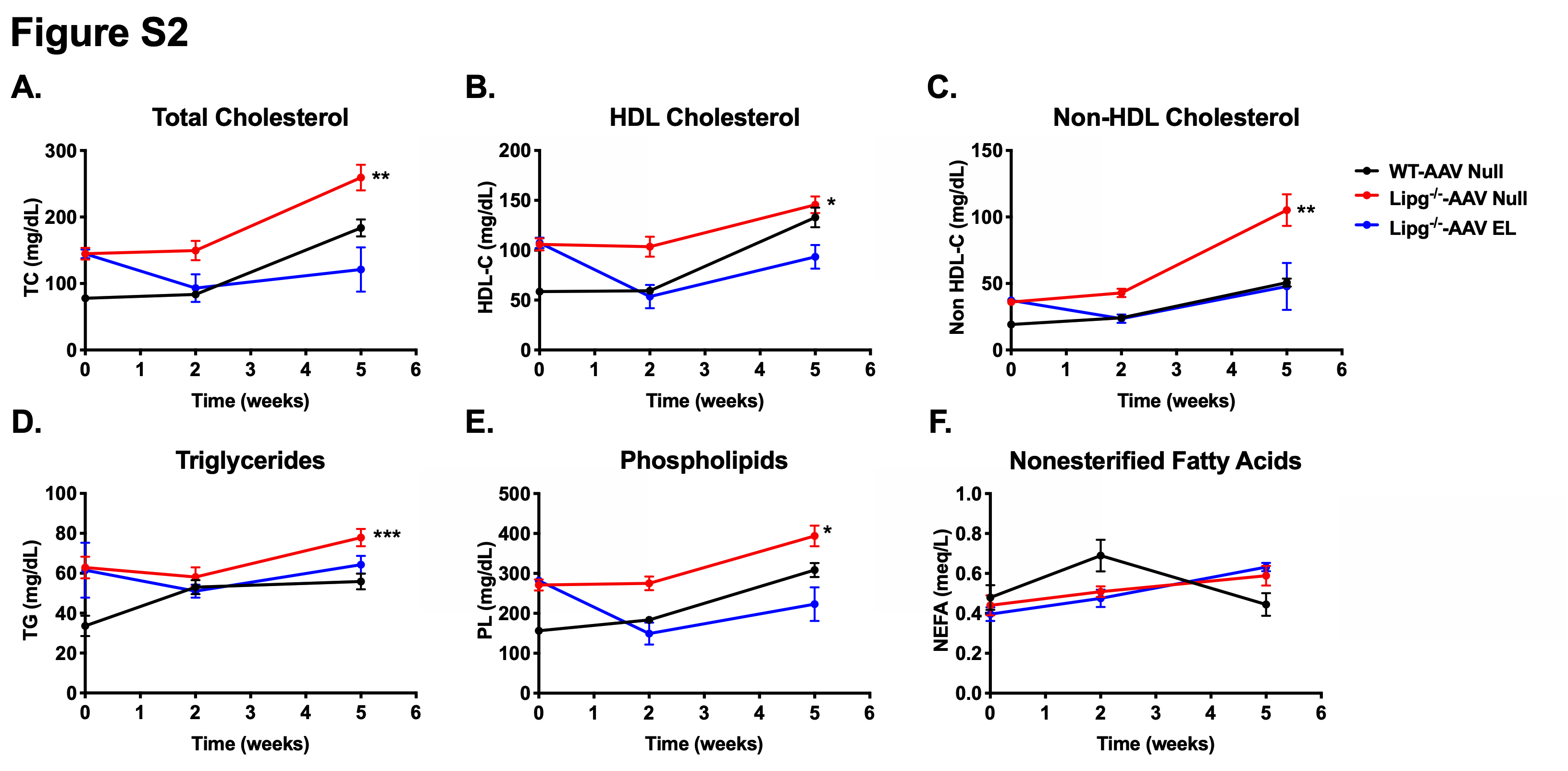

Supplement: S2 Fig — A-F. Plasma cholesterol (A), HDL-C (B), nonHDL-C (C), triglycerides (D), phospholipids (E), and nonesterified fatty acids (F) in WT mice treated with AAV Null, Lipg-/- mice treated with AAV Null, and Lipg-/- mice treated with AAV murine Lipg for 5 weeks after 4 weeks of feeding a high fat diet. Plasma was collected after 4 hours of fasting and lipids were measured by autoanalyzer. *P<0.05, **P<0.01, ***P<0.001, repeated measure 2-way ANOVA compared to the WT AAV Null group. Data is expressed as mean ± S.E.M. (TIF) [file pgen.1009802.s002.tif]
