# Supplementary material for: Endothelial lipase mediates efficient lipolysis of triglyceride-rich lipoproteins
Source: PLoS Genet. 2021 Sep 20;17(9):e1009802. doi: 10.1371/journal.pgen.1009802 (PMC8483387; doi:10.1371/journal.pgen.1009802)
Supplement: Attachment — Submitted filename: PGENETICS-D-21-00599_Reviewer_Response 07-18-21_FINAL.docx [file pgen.1009802.s006.docx]

**PGENETICS-D-21-00599 Author Response Letter**

**Response to Reviewer Comments**

**Reviewer #1**: **Khetarpal et al. have performed a detailed study of the role of endothelial lipase (EL) in the catabolism and clearance of triglyceride-rich lipoproteins (TRL). This is a nice study, with strong supporting human genetic data from meta-analyses of data from large consortia. The in vivo EL loss-of-function data showing increased plasma TG and reduced clearance of TRL from the circulation are convincing. However, the data supporting the hypothesis that EL and LPL collaborate synergistically to enhance catabolism of TRL are somewhat weak. My specific comments are detailed below.**

We appreciate the Reviewer’s enthusiasm for our work and demonstration for an important role of endothelial lipase in TG-rich lipoprotein metabolism. We agree with the Reviewer that the original manuscript, while supporting a role for EL in TG metabolism in humans and murine models, did not convincingly demonstrate that EL and LPL act collaboratively to more efficiently clear TG-rich lipoproteins. In the responses to the detailed specific queries below, we outline revisions to the manuscript that sought to address this question. Namely, we demonstrate that in vitro, using isolated and ^3^H-triolein labeled human VLDLs as substrates, EL and LPL together promoted increased triglyceride lipase activity than LPL alone in a manner that was enhanced by the addition of EL despite its relatively weaker TG-lipase activity (see reply to Question 5 below and Figure 6I of the revised manuscript). We also clarify insights from *in vitro* lipase assays studying the p.Ser169Ala variant, which we have described previously as a dominant negative variant with respect to EL activity [1]. The impact of this variant in reducing total TG lipase activity in concert with LPL (Figure 6H in revised manuscript) suggests that EL and LPL may collaborate in their promotion of TG-rich lipoprotein catabolism. Furthermore, we show *in vivo* that inhibition of LPL activity through somatic viral vector-mediated overexpression of ANGPTL4 in *Lipg*^-/-^ mice further exacerbates postprandial TGs beyond that seen in *Lipg*^-/-^ mice or ANGPTL4-overexpressing *Lipg* WT mice alone and exceeds the relative increases seen in postprandial TGs in either of these single lipase knockout or inhibition models (Figure S3 in the revised manuscript). We believe that these additional data further support our model that EL and LPL work together to promote efficient TG-rich lipoprotein lipolysis.

**1. The association of LIPG p.Asn396Ser with plasma cholesterol, HDL-C, and LDL-C is extremely significant (p=10e-74, p=10e-199, p=10e-65), however the association with TG is somewhat weaker, although still genome-wide significant (p=10e-10). Is this because VLDL is the lipoprotein with the lowest concentration of phospholipid?**

We appreciate the Reviewer’s insightful comment that the association of *LIPG* p.Asn396Ser with plasma TGs is not as strong (as assessed by p value) as that for the association with HDL-C, LDL-C and total cholesterol. We agree that the reason for this may be that the content of phospholipids in VLDL and chylomicrons is relatively less than that for LDL-C and HDL-C (approximately 19% total phospholipid content in VLDL compared with 25% in total LDL and 23-31% in HDLs). Endothelial lipase does have some triglyceride lipase activity, as demonstrated in this study and by our group and others previously, but this is likely less active than its phospholipase activity and we posit that at least some of the TG-rich lipoprotein catabolic effects of endothelial lipase are likely physiologically linked to its phospholipid catabolism.

Another explanation for the relatively weaker association of the *LIPG* p.Asn396Ser variant is the variability of plasma triglycerides with different degrees of fasting in participants and the ascertainment differences in how lipids were included between the UKBB, GLGC and MVP studies. The UKBB plasma lipid measures were all non-fasting measurements[2], whereas fasting lipids were measured in GLGC[3]. For MVP, whether lipid measures utilized were obtained with or without fasting was not specified[4], however, it is noted that maximum values were taken for all lipid measures where multiple lipid measures were available. Given that the median ± IQR plasma TGs in MVP participants utilized for this study were 211 ± 174 (Caucasians), 179 ± 149 (African American), and 221 ± 184 (Hispanic), it seems unlikely that these measures were obtained from solely fasting samples.

We have revised our text to include discussion of this point (page 13, lines 306-312):

*Our study provides a comprehensive demonstration that the LOF p.Asn396Ser variant is also associated with elevated TGs in humans. The relatively modest association between genetic LIPG loss-of-function and TGs relative to the relationship seen with HDL-C and LDL-C may reflect the lower phospholipid content of TRLs relative to these other lipoprotein classes, and variability in TGs in fasted vs. nonfasted states, for which inclusion differed among the UKBB, GLGC, and MVP studies.*

**2. Why was there only a very modest increase in phospholipids compared to TG (Fig. 3A)? Why were the TG not affected by loss of EL in the fasted animals, unlike HDL-C and LDL-C?**

Figure 3A demonstrates the change in total plasma lipid measures in WT and *Lipg*^-/-^ mice fed a chow diet after fasting for four hours and then refeeding (with chow) for four hours to increase the circulating concentration of chylomicrons in this time and help us assess a role for EL in the physiological regulation of chylomicron lipids. Whereas VLDLs contain approximately 20% of their lipid content as phospholipids, chylomicrons contain approximately 10% phospholipids among their lipid content [5], with relatively increased concentrations of triglycerides. We thus believe that the relatively lower phospholipid content of chylomicrons in the postprandial state likely explains the lack of a significant elevation in the absolute total plasma phospholipid levels after refeeding. Importantly, the relative differences in plasma phospholipids between WT and *Lipg*^-/-^ mice are still observed. Regarding the TG content in fasting mice, we believe the relatively low levels of TGs in mice on a C57BL/6 strain fed a chow diet (which is low in fat content at approximately 4.5% and low in cholesterol content at 0.02%) likely account for the lack of significant differences in plasma TGs between WT and *Lipg*-/- mice in the fasting setting. This observation led us to provoke the mice by increasing dietary triglycerides through either refeeding with chow diet for 4 hours or through high fat diet feeding (45% fat content) for several weeks as described elsewhere in the manuscript.

**3. Can the authors address why it took 4 weeks of high fat feeding for the TG levels to diverge, when other lipid parameters were affected at baseline (Fig. 3D)? Why were the changes in LDL-C so dramatic in high fat-fed animals (Fig. 3F)?**

We suggest that because circulating plasma TGs are typically low in chow diet fed mice on a C657BL/6J strain, differences in fasting plasma TGs in this model were mitigated. We believe that the differences in WT and *Lipg*^-/-^ mice with regard to TRL catabolism required sufficient accumulation of TRLs in the circulation. In contrast, C57BL/6J mice are known to have high levels of HDL cholesterol at baseline on chow diet and more phospholipid content on HDL than relative content on VLDL and chylomicrons, thus the differences in HDL cholesterol levels at baseline are more pronounced in the WT vs. *Lipg*^-/-^ mice.

The elevation in LDL-C in the setting of high fat feeding likely reflects the critical role, as described by our group [1, 6] and others [7], of endothelial lipase on LDL metabolism. In C57BL/6 J mice fed a chow diet, levels of non-HDL cholesterol are relatively low, with the predominant carrier of cholesterol in the circulation being HDL-C. We have previously shown that in situations of elevated non-HDL cholesterol through genetic deficiency of apoE or the LDL receptor or transgenic overexpression of apolipoprotein B, EL overexpression through viral vector mediated hepatic expression reduces apoB-containing lipoproteins in vivo due to its phospholipase activity[1]. More recently, we and others have shown that EL may mediate a LDL receptor-independent pathway for LDL clearance in vivo in a manner that is “masked” by ANGPTL3 inhibition of EL and could be derepressed through antibody-mediated or siRNA-mediated inhibition of ANGPTL3[6, 7]. These data support the notion that EL is a critical regulator of LDL-C catabolism. Our data in this manuscript presents complementary novel findings that deficiency of EL through genetic loss-of-function in humans and in mice in which LDL-C levels are augmented through elevated dietary fat abrogates clearance of LDL-C in addition to TRLs and HDLs.

We have included discussion of this point in our revised manuscript Discussion section (pages 14-15, lines 341-361):

*In addition to its role in TG-rich lipoprotein catabolism, our findings her demonstrate both through the human genetics and in mice that EL is critical for LDL metabolism as well. We demonstrated through our murine loss-of-function model and marked increase in LDL-C in Lipg^-/-^ mice particularly after high fat diet feeding. This extends prior work from our laboratory that demonstrated LDL as a biochemical substrate for EL-mediated phospholipid lipolysis in vitro [8, 9] and further support from our group through viral vector mediated expression of LIPG in mice showing that EL catabolizes LDL-phospholipids in vivo to promote LDL turnover [1]. That prior work demonstrated that EL overexpression in multiple models of increased circulating LDL (LDLR-deficiency, apoE deficiency, or transgenic overexpression of apolipoprotein B) increased LDL catabolism, and our findings here in Lipg^-/-^ mice in which apoB-containing lipoproteins were increased through dietary cholesterol and saturated fat elevation are complementary to that prior work. Consistent with this, another group examining apoB-containing lipoprotein catabolism in rabbits with transgenic overexpression of human LIPG observed increased apoB-containing lipoprotein turnover through increased EL-mediated phospholipase activity [10]. More recently, we and others have shown that EL may mediate a LDL receptor-independent pathway for LDL clearance in vivo in a manner that is “masked” by ANGPTL3 inhibition of EL and could be derepressed through antibody-mediated or siRNA-mediated inhibition of ANGPTL3[6, 7]. These data support the notion that EL is a critical regulator of LDL-C catabolism. Our data here extends the notion that EL facilitates further remodeling of a spectrum of apoB containing lipoproteins including large TRLs to allow more efficient TG lipolysis by LPL and thus physiologically promotes clearance in vivo.*

**4. Fig. 5B, left hand graph has groups labeled WT and Lipg–/–, whereas the right hand graph has them labeled WT and EL KO – I assume EL KO refers to Lipg–/–. These should be relabeled for consistency.**

We thank the Reviewer for this comment. We have revised the manuscript to make the designation of the endothelial lipase knockout mouse consistently as *Lipg^-/-^* throughout the manuscript and all figures.

**5. The enhancement in TG lipase activity (Fig. 6H), whilst statistically significant, is modest. As such, the data supporting the author’s hypothesis “…that EL may collaborate with LPL…” is somewhat weak.**

We agree with the Reviewer that the original version of the manuscript did not provide strong support for the notion that EL and LPL collaborate to promote lipolysis of TGs on TG-rich lipoproteins. Support for the notion that EL and LPL work together to promote TG lipolysis would come from the demonstration that addition of EL activity to LPL promotes a greater degree of TG lipolysis than the theoretical sum of either enzyme alone. We explored whether this could be true using additional models in our revised manuscript here.

We performed additional studies both in vitro and in vivo designed to support the concept of collaboration between EL and LPL in hydrolysis of TG-rich lipoproteins. In vitro, we utilized an assay to assess the impact on TG lipolysis on human TRLs by EL +/- LPL that differed from the measurement of TG lipolysis on TRL-like triolein and DMPC emulsions as shown in Figure 6 of the original manuscript. For the new assay, we isolated human VLDLs and radiolabeled them with ^3^H triolein as described in the Methods of the revised manuscript. Labeled VLDL was incubated with equivalent protein concentrations of conditioned medium containing recombinant EL, LPL or both enzymes in a solution containing 1% bovine serum albumin for 4 hours at 37 °C. Reactions were stopped with 100% ethanol, samples were incubated at -20 °C for 2 hours and then ultracentrifugated at 13,500 RPM for 20 minutes to precipitate proteins and separate lipid containing supernatants. The supernatants were run on thin layer chromatography plate to separate lipid species and fatty acids containing bands were cut and measured for ^3^H activity by scintillation counting. We found that EL and LPL each individually promoted TG lipolysis as demonstrated by our more time-limited lipase assays using TRL-like emulsions from the original version of the manuscript and included in Figure 6 E-H. As these assays on TRL-like emulsions were in part time limited to preserve the integrity of the emulsions based on our prior work, we further explored the contribution of EL and LPL individually and together to TG lipolysis on isolated human VLDLs in which reactions were carried out for 4 hours. We found that EL and LPL each individually promoted triglyceride hydrolysis on isolated human VLDLs, and that when they were combined in equal protein concentrations there was a greater degree of VLDL ^3^H triolein hydrolysis than either EL alone or LPL alone. Comparing the relative amount of TG lipolysis from the sum of that from EL alone and LPL alone to the reactions with EL and LPL combined, we found that the degree of TG lipolysis was higher in the combined reactions than that expected from just the sum of the individual enzyme reactions (P<0.05, One-sample T test comparing ^3^H oleate activity in combined lipase reactions to the mean sum of that seen for the single lipase reactions; Figure 6I in the revised manuscript, also shown below). This supports a model that EL facilitates TG lipolysis by LPL on native human VLDLs.

We also reassessed our in vitro studies (Figure 6 E-H in the original and the revised manuscript) in which we presented data on WT EL and a catalytically inactive mutant, p.Ser169Ala. Our previous work had shown that the p.Ser169Ala variant in EL is not only a catalytically inactive mutant, but also reduces the activity of WT EL in vitro and in vivo[1]. In our prior study comparing overexpression of WT and p.Ser169Ala mutant EL in mice, we showed that the mutant EL overexpression actually raised plasma phospholipids, HDL-C, nonHDL-C and triglycerides, the opposite phenotype of overexpression of adenovirus expressing WT EL. Studies of LDL-phospholipid clearance in these mice showed that p.Ser169Ala mutant EL overexpression delayed LDL-phospholipid clearance in vivo compared to WT EL overexpression and compared to mice expressing Null adenovirus. Further studies in COS7 cells stably expressing murine EL and infected with WT or p.Ser169Ala mutant EL adenovirus showed that the co-expression of murine WT and mutant EL reduced phospholipase activity relative to co-expression with murine WT and human WT EL, and that the mutant protein could not be co-immunoprecipitated with murine EL from conditioned media from the cells, suggesting that the p.Ser169Ala mutant from of EL may bind to and perturb secretion of endogenous EL. Indeed in our experiments shown in Figure 6H, we see that whereas WT EL contributes positively to the total TG lipase activity on TRL-like emulsions when added with LPL, the p.Ser169Ala mutant EL reduces total TG lipase activity (P<0.0001, two-way ANOVA, Figure 6H below and in revised manuscript). The finding that this dominant negative form of EL can reduce the TG lipase activity of LPL in vitro suggests that the normal activity of EL is to collaborate with LPL in TG lipolysis on TG-rich lipoproteins, and that negative perturbation of this activity in a manner that reduces EL-mediated TG lipolysis also reduces total TG lipolysis of these lipoproteins, even that conferred by LPL.

To see if this collaborative relationship was observed physiologically in vivo, we sought to generate a model of combined EL and LPL inactivation in mice. Given the limitations in time to obtain or breed mice with conditional LPL deficiency, we generated a model of lipase inhibition through somatic overexpression of an established inhibitor of LPL, ANGPTL4, through hepatic adeno-associated virus (AAV) expression of murine ANGPTL4 in *Lipg*^-/-^ mice. We compared fasting plasma lipids over 6 weeks after AAV ANGPTL4 administration in these mice to either *Lipg*^-/-^ mice treated with Null AAV or to WT mice treated with ANGPTL4 AAV to simulate comparisons to inhibition or loss-of-function of each individual lipase to that in the model of the combined lipase inactivation. We also compared postprandial TGs in these models after olive oil gavage. The details and results of this experiment are summarized below in reply to Reviewer 1 Query 8 and also appear as Figure S3 in the revised manuscript. We found that ANGPTL4 AAV in WT mice increased fasting and postprandial TGs as expected. We further showed that LPL inhibition through ANGPTL4 overexpression in *Lipg*^-/-^ mice further exacerbated the elevation in fasting and postprandial TGs. The relative increase in TGs after 7 hours from olive oil gavage in the *Lipg*^-/-^ mice treated with ANGPTL4 AAV were significantly higher than that for the mean sum of the *Lipg*^-/-^ mice treated with Null AAV and the WT mice treated with ANGPTL4 AAV (Figure S3 in the revised manuscript). This suggests that in vivo, EL and LPL may work together in a collaborative fashion to promote more efficient lipolysis of TG rich lipoproteins.

In summary we believe that the totality of data presented here in our revised manuscript more robustly supports the notion that EL acts collaboratively with LPL to promote TG lipolysis on TG-rich lipoproteins. We have revised our Results and Discussion to reflect this new data and conclusions.

**Figure 6 H-I from revised manuscript**

**6. The authors use the catalytic mutant p.Ser169Ala in the studies in Fig. 6. What about the effects of the human p.Asn396Ser variant? These studies would support the role of this variant in regulating phospholipase activity.**

We agree with the Reviewer that demonstration of loss of enzymatic activity by the p.Asn396Ser variant in *LIPG* would be helpful in supporting this variant as loss-of-function with respect to EL phospholipase activity. Key support for the notion that *LIPG* p.Asn396Ser is a loss-of-function variant for EL enzymatic activity comes from prior work from our group and others studying the in vitro impact of this variant on phospholipase activity on HDL or HDL-like phospholipid emulsions [11, 12]. Work from our group by Edmondson and colleagues [11] showed that recombinant human EL harboring p.Asn396Ser had approximately 25% of the phospholipase activity of WT recombinant EL on dimyristoyl-phosphatidylcholine (DMPC) lipid emulsions and human isolated HDL_3_ in vitro. We further showed in that study that adeno associated virus (AAV) vectors expressing p.Asn396Ser mutant EL in mice conferred impaired reduction in HDL-C in vivo compared to WT EL AAV. Work from Singaraja and colleagues found that recombinant EL harboring p.Asn396Ser had approximately 50% phospholipase activity on fluorescently labeled synthetic HDL-like micelles [12].

To further assess the impact of *LIPG* p.Asn396Ser on TG-rich lipoproteins, we performed an assay to assess the uptake of 3H-Triolein labeled human VLDL in transiently transfected cells expressing WT EL or the p.Asn396Ser mutation. For further comparison, we also tested another rare predicted loss-of-function (pLOF) variant in *LIPG*, p.Met342Val, that we previously reported as a rare contributor to genetically elevated HDL-C in humans and confers <20% phospholipase activity on DMPC phospholipid emulsions or HDL_3_ in vitro [11]. For this experiment, we transiently transfected HEK293-T cells with plasmids expressing WT, p.Asn396Ser, or p.Met342Val mutants of human EL, or a plasmid expressing green fluorescent protein (GFP) as previously described [11]. ^3^H-triolein labeled VLDL was generated as described above and in the Methods of our current manuscript. 24 hours after transfection, cells were incubated with ^3^H-triolein labeled VLDL (TG concentration 0.04 mg/dl) for 1 hour and following this, media was removed and cell lysates were collected in RIPA buffer and ^3^H-triolein activity was measured using scintillation counting. Results from this preliminary experiment of three replicates in each group shown in Figure R1_6 below show that there was approximately two-fold uptake of ^3^H-triolein in the cells transfected with WT EL relative to those transfected with GFP expressing plasmid. Cells expressing the EL p.Asn396Ser mutant demonstrated approximately 50% the uptake of ^3^H-triolein compared to those expressing WT EL. As shown previously for the p.Met342Val variant with regard to HDL phospholipase activity, we show here that this variant confers near complete loss-of-function with regard to ^3^H-triolein uptake from ^3^H-triolein labeled human VLDL (*P<0.05 compared with WT, Student’s unpaired T-test).

Given the nature of this experiment in which uptake from transfected HEK293-T cells is measured rather than the in vitro phospholipase activity experiments using ^3^H-triolein labeled TRL-like emulsions and recombinant EL proteins from media of large quantities of transfected cells, as is shown in Figure 5 of the manuscript, we did not include the data below in Figure R1_6 in the revised manuscript. However we believe these data supports the already published data using a similar in vitro assay using phospholipid emulsions by our group and others that the p.Asn396Ser variant of *LIPG* confers likely 50% or more loss of phospholipase activity on lipoprotein phospholipids including HDLs and TG-rich lipoproteins. We have revised our manuscript to include mention of the prior work demonstrating that p.Asn396Ser confers loss of function of phospholipase activity.

**7. As the authors state that “…EL is expressed throughout the vascular endothelium where LPL is located…” and “…that EL may collaborate with LPL…”, are there any humans with LOF variants in both LPL and LIPG?**

This is an excellent question and we agree with the Reviewer that humans with combined genetic loss-of-function in both endothelial lipase and lipoprotein lipase would be incredibly informative into the phenotypic consequences of impaired extracellular lipase activity, both with regards to plasma lipid metabolism but other broader phenotypes. In a limited investigation of a subset of the UK BioBank participants who underwent whole exome sequencing for whom we had annotated predicted loss-of-function (pLOF) variants in both *LPL* and *LIPG* (N=49,959 in total), we identified 156 participants who carried one or more copy of a pLOF variant in *LIPG*, 92 participants with one or more copy of a pLOF variant in *LPL*, but unfortunately no participants in that subset who carried one or more copy of a pLOF variant in both genes. Efforts by our group are underway to assess this question in additional, larger cohorts, including the expanded set of whole exomes from the UK BioBank, and cohorts in which there is consanguinity and a potential enrichment for rare pLOF variants such as the Pakistan Risk Of Myocardial Infarction Study (PROMIS; [13]). We have included mention of the lack of finding any human carriers of pLOF variants in both lipase genes in the subset of the UK BioBank studied in our revised Discussion.

**8. Loss-of-function studies in mice lacking both LPL and EL would strengthen the study.**

We agree with the Reviewer that insights about the relationship of EL and LPL with respect to TRL TG catabolism would be greatly informed by murine studies of combined EL and LPL deficiency. To that end, we studied the impact of inhibition of LPL in *Lipg*^-/-^ mice through somatic hepatic overexpression of ANGPTL4, a well-established endogenous circulating inhibitor of LPL activity in mice and humans[14]. We used an adeno-associated virus (AAV) vector expressing murine ANGPTL4[15] in WT and *Lipg*^-/-^ mice (6-8 mice per group) and compared to expression of a control AAV vector expressing the LacZ gene. The results from this experiment are shown below and also appear as Figure S3 in the revised manuscript.

As supported by other data presented in our study, *Lipg*^-/-^ mice expressing Null AAV demonstrated significantly elevated plasma HDL cholesterol, nonHDL cholesterol, and phospholipids, and a trend towards elevated fasting triglycerides relative to WT mice expressing Null AAV(*, P<0.05 compared with WT-AAV Null group, Figure S3 A-D). The expression of ANGPTL4 AAV in *Lipg*^-/-^ mice did not further elevate plasma HDL cholesterol, nonHDL cholesterol and phospholipids in the fasted state over this timeframe (Figure S3 A-C). However we did observe a significant increase in fasting triglycerides in WT mice (as previously shown through LPL inhibition with ANGPTL4 AAV expression in mice[15]), and a further elevation in fasting triglycerides in *Lipg*^-/-^ mice expressing ANGPTL4 AAV relative to either *Lipg*^-/-^ mice expressing Null AAV or WT mice expressing ANGPTL4 AAV (Figure S3 D).

We further challenged the mice with oral fat tolerance testing to measure postprandial triglycerides. Triglycerides 7 hours after olive oil gavage in the AAV Null treated *Lipg*^-/-^ mice and in WT mice expressing ANGPTL4 AAV were elevated above that of AAV Null treated WT mice. Triglycerides in the *Lipg*^-/-^ mice expressing ANGPTL4 AAV were further elevated (Figure S3 E). When we compared the relative increase in post gavage plasma triglycerides compared with the Null treated WT mice (Figure S3 F), we found that the increase in the ANGPTL4-expressing *Lipg*^-/-^ mice was significantly greater than the mean sum of the increase (140.0%, shown in the red dotted line) in the AAV Null expressing *Lipg*^-/-^ mice and the AAV ANGPTL4 expressing WT mice (P<0.001, one-sample T-test). While this is a limited investigation of one model of LPL inhibition, these results suggest that LPL inhibition in the setting of murine EL deficiency may further increase plasma triglycerides, and that in the postprandial setting this may be further exacerbated.

**Figure S3: Impact of LPL inhibition with ANGPTL4 overexpression in *Lipg*^-/-^ mice. A-D.** Plasma HDL-C, nonHDL-C, phospholipids, and TGs from WT vs. *Lipg*^-/-^ mice treated with AAV Null vector (WT-AAV Null; *Lipg*^-/-^ - AAV Null) or AAV ANGPTL4 vector (WT-AAV ANGPTL4; *Lipg*^-/-^ - AAV ANGPTL4) after a 4 hour fast. Lipids were measured by chemical autoanalyzer. **E-F**. Plasma TGs 7 hours after oral fat tolerance testing in mice from (A). TGs in mg/dl are shown in (E). The relative difference in percent (%) increase above the WT – AAV Null group in plasma TGs from each group in (E) is shown in (F). Red dotted line shows the mean summed % increase in 7 hour plasma TGs for the *Lipg*^-/-^ - AAV Null and the WT-AAV ANGPTL4 group. For A-D, *, P<0.05, 2-way ANOVA comparing indicated group to WT-AAV Null group. For E-F, *P<0.05, **P<0.01, ***P<0.001, student’s unpaired T-test comparing indicated group to WT – AAV Null group. In (F), also shown for *Lipg*^-/-^ - AAV ANGPTL4 is significance for one-sample T-test comparing this group to the mean summed % increase in TG for WT-AAV ANGPTL4 and *Lipg*^-/-^ - AAV Null groups (140.0), ***P<0.001). All error bars indicate mean ± S.E.M.

**Reviewer #2**:

**1. This is an important study demonstrating for the first time the role of EL in TLR catabolism. The authors provide compelling human and mouse data implicating EL in plasma TG metabolism. The manuscript is well written, the experiments are well designed and an impressive amount of high quality results is clearly and rigorously presented.**

**A major contention of the study is that EL facilitates TG lipolysis in concert with LPL and the two enzymes act synergistically. However, this conclusion is not supported by the data provided. EL has TG lipase activity (Fig. 6E) and it clearly acts additively with LPL activity (Fig. 6H). Thus, there appears to be no synergism between the two enzymes and the model of EL’s phospholipase activity promoting TG lipolysis by LPL is not born out by the in vitro data. Furthermore, the accumulation of PUFA-containing TGs in EL deficiency (Fig. 6B) suggests that it is EL’s TG lipase activity that is responsible for elevated plasma TG in vivo. Thus, contrary to the model shown in Fig. 7, this study seems to demonstrate that EL contributes to TRL metabolism through its TG lipase activity. The manuscript should be revised accordingly.**

We greatly appreciate the Reviewer’s enthusiasm for our work and the conclusions with regard to EL and TG-rich lipoproteins presented in this manuscript.

We agree with the Reviewer that our original manuscript suggests that EL has a direct impact on TG lipolysis and that our data did not demonstrate that it is the phospholipase activity of EL that promotes TG lipolysis on these lipoproteins. Ultimately the notion that the phospholipase activity of EL facilitated TG lipolysis by LPL was supported by prior work demonstrating that the predominant activity of EL is phospholipase activity and that this is observed in vitro on TG-rich lipoproteins as well[9]. Further support for the phospholipase activity of EL being important in apoB-containing lipoprotein catabolism came from studies of EL overexpression demonstrating an important role in LDL catabolism and LPL-phospholipid turnover in vivo[1]. In vitro assays comparing relative phospholipase to TG lipase activity of EL and LPL against radioisotope labeled lipid emulsions found that EL has approximately ~215 times the phospholipase:TG-lipase activity ratio as LPL [9]. For this reason, we proposed that given our demonstration of robust phospholipase activity of EL on TG-rich lipoproteins and the comparatively weaker TG lipase activity on these lipoproteins, particularly in comparison to the preeminent TG lipase LPL on these same particles, the phospholipase activity of EL would be the predominant contribution to the TG lipase activity by LPL in promoting particle turnover.

Ultimately, because the hydrolysis of TGs and phospholipids by EL is thought to be due to the same catalytic activity, mutagenesis studies alone would be insufficient in helping to dissect these activities and determine their relative contribution. No EL variants have been reported that selectively affect PLase vs TGase activity. We surmise that one way to test the contribution of EL’s phospholipase activity to TG lipolysis on TG-rich lipoproteins would be to generate TRL-like emulsions containing non-hydrolyzable phospholipids as an outer shell on hydrolysable TGs and test the ability of EL to promote TG hydrolysis alone or in concert with LPL on such particles. If EL is able to promote TG lipolysis alone or in concert with LPL on such particles, it would further suggest that EL harbors potent and likely underappreciated TG lipase activity itself independently of its phospholipase activity. Alternatively, if EL cannot promote TG lipolysis alone, or if LPL lipolysis of TGs on such TRL-like emulsions is blunted by the impaired ability of EL to hydrolyze phospholipids on these particles, it would suggest that the phospholipase activity of EL promotes TG lipolysis by LPL on these particles. Unfortunately, generation of such TRL-like emulsions and execution of such an experiment is beyond the scope of this manuscript. We have thus revised our manuscript to soften our conclusions a bit and acknowledge that further experiments will be needed to more completely dissect the relative contributions of the phospholipase and TG lipase activities of EL on TG-rich lipoprotein hydrolysis.

With regard to the question of whether EL acts additively or synergistically to promote TG lipolysis with LPL, we agree with the Reviewer that the originally submitted version of the manuscript had limited data to suggest that EL’s contribution to TG lipolysis was formally synergistic with LPL. As discussed in reply to Reviewer 1 Question 5 above in this Response Letter in which Reviewer 1 made a similarly important point about the lack of support for the suggestion of a collaborative contribution of EL to TG lipolysis, we have worked to further substantiate this claim in our revised manuscript. First, we show through in vitro lipase assays comparing TG lipolysis on intact human VLDLs in which lipolysis is allowed to continue for a duration of 4 hours that EL and LPL contribute to a significantly higher degree of TG lipolysis on VLDLs than would be expected from the sum of the TG hydrolysis from either enzyme alone (Figure 6I in the revised manuscript, also shown above in reply to Reviewer 1 Question 5). Next we note that the catalytically inactive variant in EL, p.Ala169Ser, that was compared to WT EL in the in vitro lipase assays against TG-rich lipoprotein like lipid emulsions in Figure 6 E-H in the original manuscript has been previously shown by our group to be a dominant negative variant with regard to EL phospholipase activity likely through forming heterodimers with EL and preventing secretion and co-immunoprecipitation with endogenous EL and reducing phospholipase activity in vitro and in vivo. Comparison of the impact of the p.Ala169Ser mutant to WT EL on total TG lipolysis along with LPL in Figure 6I (shown above in reply to Reviewer 1 Question 5) shows that this dominant negative mutant reduces total TG lipolysis. We conclude EL and LPL act in concert in vitro based on the finding that a dominant negative EL mutant reduces the TG lipolysis conferred by LPL. Finally, in vivo we explored the relationship of combined inhibition of EL and LPL through overexpression of the LPL inhibitor ANGPTL4 in *Lipg*^-/-^ mice (see reply to Reviewer 1 Question 8 and Figure S3 shown in that reply and in the revised manuscript). We demonstrate that reduction in TG lipolysis of postprandial TG-rich lipoproteins is exacerbated in the setting of combined lipase inhibition (ANGPTL4 AAV-mediated overexpression in *Lipg*^-/-^ mice) compared to the inhibition or deficiency of either lipase alone (AAV Null treated *Lipg*^-/-^ mice or AAV ANGPTL4 treated WT mice). This impact appears to be significantly more than then sum total of the relative increases in postprandial TGs from loss of function of either lipase alone (Figure S3 F). Due to these findings, we conclude that EL collaboratively or synergistically facilitates TG lipolysis on TRLs along with LPL and have revised our manuscript to reflect this new data and conclusions.

We have revised our discussion of the potential mechanisms by which EL may facilitate TG lipolysis, limitations in the ability from our data to dissect the relative contributions of phospholipase activity with TG lipase activity on TRLs, and the fact that our data at least demonstrably shows that EL has significant TG lipase activity on TRLs that collaboratively increases total TG lipase activity with LPL on these lipoproteins in the revised manuscript in our Discussion section (page 14, lines 327-341):

*The mechanisms by which EL contributes to this may include both its phospholipase activity, which is thought to be its predominant lipase activity, and also underappreciated direct TG lipolysis. Here we show in our in vitro experiments and in lipidomic characterization of postprandial TRLs from Lipg-/- mice that loss-of-function of EL impairs both phospholipid and TG lipolysis on TRLs. Since TRLs possess a phospholipid shell surrounding a neutral lipid TG core and our data and prior work suggests that EL possesses significantly higher phospholipase:TG lipase activity on a spectrum of lipoproteins, we hypothesize that one mechanism by which EL confers more efficient TG lipolysis along with LPL is through “unshielding” the exterior PCs through its phospholipase activity and contributing to initial TG lipolysis to allow more substantive TG lipolysis subsequently by LPL. Our data presented here is limited in its ability to dissect the relative contributions of phospholipase vs TG lipase activities of EL to determine which predominantly impacts the ability of EL to promote TRL-TG lipolysis. Our work does demonstrate that EL has underappreciated TG lipase activity on TRLs and that this likely contributes at least in part to the markedly enhanced TRL lipolysis seen in concert with LPL in vitro and in vivo.*

**Minor comments:**

**2. Fig. S1: According to the figure legend, one-way ANOVA was used to analyze data in S1A. This is likely to be a typo, as repeated measure two-way ANOVA should have been used. Also, the calculation of slopes in S1B is unclear. According to Methods, the 0, 60 and 240 min time-points were used. However, the curve is clearly not linear at across this time span. It would be more appropriate to use the 60, 120 and 240 time points for slope estimation.**

We thank the Reviewer for this comment. The analysis of the TG secretion-time curves in Figure S1A was indeed completed using repeated measure two-way ANOVA, not one-way ANOVA as erroneously described in the original version of the manuscript. We have revised the manuscript here to correct this. We have also included the timepoints of 60, 120, and 240 minutes in the slope calculation of Figure S1B in our revised manuscript. We include the revised Figure S2 below.

**Figure S1: VLDL-TG secretion in *Lipg^-/-^* mice. A.** Plasma triglycerides in mice at the indicated timepoints following intraperitoneal injection of poloxamer P407, a competitive inhibitor of LPL activity. Plasma TGs were measured by colorimetric biochemical assays. **B.** VLDL-TG secretion rates calculated from the slope of the curves from (A) using the 1, 2, and 4 hour timepoints. **P<0.01, repeated measured two-way ANOVA comparing WT and *Lipg^-/-^* groups (A). *P<0.05, student’s unpaired T-test comparing WT and *Lipg^-/-^* groups (B). Data is expressed as mean ± S.E.M.

Fig. 3, Page 8: The authors claim that the mouse HF data suggest “delayed clearance of remnant TRLs in the absence of EL function”. It is unclear what points to reduced remnant clearance. These data seem to indicate reduced TRL clearance.

**3. Fig. S2: Fasting TG levels before AAV injection are almost double in EL-KO vs WT mice in this experiment. This is in sharp contrast to data in Fig. 3A, where no difference is seen. The apparently contradicting results should be explained.**

We thank the Reviewer for pointing this out. The AAV overexpression of EL in *Lipg*^-/-^ mice vs WT mice was completed after 4 weeks of feeding the mice a high fat diet as described for the experimental comparisons of WT and *Lipg*^-/-^ mice on the same high fat diet shown in Figure 3 D-F and Figure 4 C-D. At approximately 4 weeks of 45% kcal high fat diet feeding, the *Lipg*^-/-^ mice start to demonstrate modest elevations in fasting TG relative to WT mice fed the same diet. We have amended the Results and Methods to reflect this.  **4. Fig. S2: The meaning of double diamonds is unclear. The figure legend states that these indicate T-test significance between AAV treatments. This is clearly not the case and should be clarified. All time course data, including WT vs EL-KO should be analyzed the same way, preferably by repeated measure two-factor ANOVA.**

We thank the author for guidance in revising our manuscript’s statistical analyses and have revised Figure S2 to include comparisons made by repeated measure 2-way ANOVA with comparison to the WT AAV Null group. The figure and figure legend have been revised accordingly.

**Figure S2: Reconstitution of EL in the liver of *Lipg^-/-^* mice.** A-F. Plasma cholesterol (A), HDL-C (B), nonHDL-C (C), triglycerides (D), phospholipids (E), and nonesterified fatty acids (F) in WT mice treated with AAV Null, *Lipg*^-/-^ mice treated with AAV Null, and Lipg *Lipg*^-/-^ mice treated with AAV expressing *LIPG* for 5 weeks after 4 weeks of feeding a high fat diet. Plasma was collected after 4 hours of fasting and lipids were measured by autoanalyzer. *P<0.05, **P<0.01, ***P<0.001, repeated measure 2-way ANOVA compared to the WT AAV Null group. Data is expressed as mean ± S.E.M.

**5. Fig. 4 and 5: Again, it is unclear how one-way ANOVA, as stated in the figure legend was used in 4A and 4C. Two-way ANOVA should be used to analyze all time course data including those in 4E. The same comment applies to Fig. 5A and 5C. In 5E, LPL data are clearly not normally distributed. Non-parametric analysis should be used instead of T-test.**

We thank the Reviewer for this comment and have revised our statistical analysis throughout the manuscript such that all time course data is now analyzed using repeated factor 2-way ANOVA. We have carefully reviewed that the following data panels in our revised manuscript reflect this revised analysis in the text, figure panels and figure legends.

Figure 3 D; Figure 4 A, C and E; Figure 5 A and C; Figure 6 E-H; Figure S1 A; Figure S2 A-F; Figure S3 A-D **6. Fig. 5, Page 9: Again, it is unclear why the authors “hypothesized the delay in remnant TRL clearance”, when it is TG hydrolysis that is affected in EL deficiency.**

Based on the data we presented in Figure 4 from oral fat tolerance testing in WT or *Lipg*^-/-^ mice in the setting of chow or high fat diet feeding, we concluded that there was a delay in the clearance of chylomicron particles as evidenced by elevated TRL TGs at the 7 hour timepoints in Figures 4A and C. Our data in Figure 5 measuring lipolysis of 3H triolein labeled TRLs administered intravenously in our mouse model further suggests that the mechanism behind this delay in clearance is defective TG lipolysis. However, the data in Figure 4 demonstrate that there is a persistent elevation in TRL TGs hours after oral fat loading, suggesting that the impaired lipolysis of TGs in *Lipg*^-/-^ mice causes a delay in clearance of these particles. Further support for there being delayed particle clearance comes from immunoblotting FPLC fractionated plasma 7 hours after oral fat loading in the high fat diet fed WT or *Lipg*^-/-^ mice for apolipoprotein B (shown in Figure R2_6 below). FPLC fractions used for measurement of TG and cholesterol as shown in Figure 4 D was subjected to SDS-PAGE and immunoblotted for murine apolipoprotein B. One can see prominent apoB100 and apoB48, suggestive of chylomicron and chylomicron remnants, in the fractions corresponding to TG-rich lipoproteins from the 7 hour timepoint in the *Lipg*^-/-^ fractionated plasma but not in the WT group plasma.

Delay in chylomicron particle and remnant clearance may be due solely due to impaired TG lipolysis as our data has shown in other parts of the manuscript, or impaired bridging of lipoproteins to lipoprotein receptors by EL in its absence. Bridging of lipoproteins and remnants for hepatic uptake is a phenomenon of the extracellular lipases including EL that we have explored previously. We did not assess the contribution of this putative role for EL in lipoprotein clearance in this manuscript and have acknowledged this in our Discussion.

**7. Fig. 7: Most FA released by LPL (and likely EL) action is taken up by the tissues where hydrolysis takes place (i.e. muscle, adipose). Showing FFA exclusively taken up by liver is misleading.**

We agree with the Reviewer that given the role of extrahepatic tissues in the uptake of postprandial TG-derived fatty acids after lipolysis, it important to measure the uptake from the ^3^H triolein TRL lipolysis experiment in other tissues. In addition to the liver, we measured ^3^H activity after the plasma clearance in inguinal white adipose tissue, brown adipose tissue, heart, spleen and skeletal muscle (gastrocnemius) from each mouse and normalized tissue ^3^H activity in a fixed sample of tissue to the activity from the 1 minute plasma sample. We found that on a mass basis there was higher uptake of ^3^H activity in the liver compared to other tissues, likely reflecting the clearance of TRL particles by the liver itself. There was a smaller but statistically significant delay in uptake of ^3^H oleate into brown adipose tissue, and a higher uptake of ^3^H activity in the spleen of the *Lipg*^-/-^ mice, likely reflecting splenic macrophage clearance of chylomicrons as has previously been described [16]. We include this data in place of the prior data showing only the hepatic ^3^H uptake in Figure 5D of the revised manuscript and below. We have also revised our schematic diagram summarizing our work in Figure 7 to reflect this important point made by the Reviewer (please see Figure 7 in the revised manuscript, also included below in reply to Reviewer 3 Question 1).

**Figure 5D**

Uptake of ^3^H-oleate into the indicated tissues after 15 min of administration of ^3^H-triolein labeled TRLs in the mice from (A). ^3^H activity in a fixed amount of tissue was normalized to the 1 minute plasma ^3^H activity for each mouse. *P<0.05, **P<0.01, student’s unpaired T-test.

**8. Discussion: pLOF should be defined.**

We have defined this in the Keywords section of the manuscript.

**Reviewer #3:**

**Dear Authors,
I congratulate you all on a nice study blending human genetics with biochemical characterization of endothelial lipase. One aspect of the manuscript that could use clarification is uptake of fatty acids by the liver. In its current written format it comes across that there is impairment of fatty acid uptake. I believe what you mean is that EL allows for cleavage of particle followed by uptake of oleate in the liver. This could be amended in the results and discussion to read clear.**

We thank the Reviewer for the kind enthusiasm and support of our work. We very much appreciate and agree with the suggestion from the Reviewer. We have revised our manuscript to indicate that our data supports EL in playing a role in TG lipolysis and TG-rich lipoprotein clearance but not that EL impacts hepatic fatty acid uptake itself. We believe that our data supports the notion that through impairment of TG lipolysis, loss of function of EL impairs TG-rich lipoprotein remnant formation and hepatic clearance of these remnants, rather than fatty acid uptake mechanisms by the liver itself. We have revised our manuscript and our schematic in Figure 7 to reflect this as shown now below.

**References**

1.	Broedl UC, Maugeais C, Millar JS, Jin W, Moore RE, Fuki IV, et al. Endothelial lipase promotes the catabolism of ApoB-containing lipoproteins. Circulation research. 2004;94(12):1554-61. Epub 2004/05/01. doi: 10.1161/01.res.0000130657.00222.39. PubMed PMID: 15117821.

2.	Van Hout CV, Tachmazidou I, Backman JD, Hoffman JD, Liu D, Pandey AK, et al. Exome sequencing and characterization of 49,960 individuals in the UK Biobank. Nature. 2020;586(7831):749-56. Epub 2020/10/23. doi: 10.1038/s41586-020-2853-0. PubMed PMID: 33087929; PubMed Central PMCID: PMCPMC7759458.

3.	Liu DJ, Peloso GM, Yu H, Butterworth AS, Wang X, Mahajan A, et al. Exome-wide association study of plasma lipids in >300,000 individuals. Nature genetics. 2017;49(12):1758-66. Epub 2017/10/31. doi: 10.1038/ng.3977. PubMed PMID: 29083408; PubMed Central PMCID: PMCPMC5709146.

4.	Klarin D, Damrauer SM, Cho K, Sun YV, Teslovich TM, Honerlaw J, et al. Genetics of blood lipids among ~300,000 multi-ethnic participants of the Million Veteran Program. Nature genetics. 2018;50(11):1514-23. Epub 2018/10/03. doi: 10.1038/s41588-018-0222-9. PubMed PMID: 30275531; PubMed Central PMCID: PMCPMC6521726.

5.	Gropper SSS, J. L.; Groff, J. L. . Advanced Nutrition and Human Metabolism: Wadsworth Publishing; 2008.

6.	Wu L, Soundarapandian MM, Castoreno AB, Millar JS, Rader DJ. LDL-Cholesterol Reduction by ANGPTL3 Inhibition in Mice Is Dependent on Endothelial Lipase. Circulation research. 2020;127(8):1112-4. Epub 2020/08/19. doi: 10.1161/CIRCRESAHA.120.317128. PubMed PMID: 32808882.

7.	Adam RC, Mintah IJ, Alexa-Braun CA, Shihanian LM, Lee JS, Banerjee P, et al. Angiopoietin-like protein 3 governs LDL-cholesterol levels through endothelial lipase-dependent VLDL clearance. Journal of lipid research. 2020;61(9):1271-86. Epub 2020/07/11. doi: 10.1194/jlr.RA120000888. PubMed PMID: 32646941; PubMed Central PMCID: PMCPMC7469887.

8.	Brown RJ, Miller GC, Griffon N, Long CJ, Rader DJ. Glycosylation of endothelial lipase at asparagine-116 reduces activity and the hydrolysis of native lipoproteins in vitro and in vivo. Journal of lipid research. 2007;48(5):1132-9. Epub 2007/02/27. doi: 10.1194/jlr.M600535-JLR200. PubMed PMID: 17322565.

9.	McCoy MG, Sun GS, Marchadier D, Maugeais C, Glick JM, Rader DJ. Characterization of the lipolytic activity of endothelial lipase. Journal of lipid research. 2002;43(6):921-9. Epub 2002/05/29. PubMed PMID: 12032167.

10.	Wang C, Nishijima K, Kitajima S, Niimi M, Yan H, Chen Y, et al. Increased Hepatic Expression of Endothelial Lipase Inhibits Cholesterol Diet-Induced Hypercholesterolemia and Atherosclerosis in Transgenic Rabbits. Arteriosclerosis, thrombosis, and vascular biology. 2017;37(7):1282-9. Epub 2017/05/27. doi: 10.1161/ATVBAHA.117.309139. PubMed PMID: 28546217; PubMed Central PMCID: PMCPMC5513187.

11.	Edmondson AC, Brown RJ, Kathiresan S, Cupples LA, Demissie S, Manning AK, et al. Loss-of-function variants in endothelial lipase are a cause of elevated HDL cholesterol in humans. The Journal of clinical investigation. 2009;119(4):1042-50. Epub 2009/03/17. doi: 10.1172/jci37176. PubMed PMID: 19287092; PubMed Central PMCID: PMCPMC2662558.

12.	Singaraja RR, Sivapalaratnam S, Hovingh K, Dube MP, Castro-Perez J, Collins HL, et al. The impact of partial and complete loss-of-function mutations in endothelial lipase on high-density lipoprotein levels and functionality in humans. Circ Cardiovasc Genet. 2013;6(1):54-62. Epub 2012/12/18. doi: 10.1161/CIRCGENETICS.111.962613. PubMed PMID: 23243195.

13.	Saleheen D, Natarajan P, Armean IM, Zhao W, Rasheed A, Khetarpal SA, et al. Human knockouts and phenotypic analysis in a cohort with a high rate of consanguinity. Nature. 2017;544(7649):235-9. Epub 2017/04/14. doi: 10.1038/nature22034. PubMed PMID: 28406212; PubMed Central PMCID: PMCPMC5600291.

14.	Basu D, Goldberg IJ. Regulation of lipoprotein lipase-mediated lipolysis of triglycerides. Curr Opin Lipidol. 2020;31(3):154-60. Epub 2020/04/26. doi: 10.1097/MOL.0000000000000676. PubMed PMID: 32332431; PubMed Central PMCID: PMCPMC7478854.

15.	Lei X, Shi F, Basu D, Huq A, Routhier S, Day R, et al. Proteolytic processing of angiopoietin-like protein 4 by proprotein convertases modulates its inhibitory effects on lipoprotein lipase activity. The Journal of biological chemistry. 2011;286(18):15747-56. Epub 2011/03/15. doi: 10.1074/jbc.M110.217638. PubMed PMID: 21398697; PubMed Central PMCID: PMCPMC3091183.

16.	Crawford SE, Borensztajn J. Plasma clearance and liver uptake of chylomicron remnants generated by hepatic lipase lipolysis: evidence for a lactoferrin-sensitive and apolipoprotein E-independent pathway. Journal of lipid research. 1999;40(5):797-805. Epub 1999/05/01. PubMed PMID: 10224148.
