# Supplementary material for: Endothelial lipase mediates efficient lipolysis of triglyceride-rich lipoproteins
Source: PLoS Genet. 2021 Sep 20;17(9):e1009802. doi: 10.1371/journal.pgen.1009802 (PMC8483387; doi:10.1371/journal.pgen.1009802)
Supplement: Attachment — Submitted filename: PGENETICS-D-21-00599R1_Reviewer_Response_08-25-21_FINAL.docx [file pgen.1009802.s007.docx]

**PGENETICS-D-21-00599R1 Author Response Letter**

**Response to Reviewer Comments**

**Reviewer #2**:

**The authors addressed the reviewers’ questions in detail and substantially revised and improved the manuscript. Importantly, they included new data that demonstrate the interaction between EL and LPL in TRL hydrolysis in vitro and in vivo. Addressing the following issues would further strengthen the manuscript:**

**1. Line 245: Reference to Fig. 6G is missing.**

We agree with the Reviewer and have added reference to Figure 6G on Line 245 of the manuscript.

**2. Line 246: The reference to 30 min is confusing, as EL increases TG-lipase activity up to 100 min.**

The in vitro lipase assays using recombinant lipases with ^3^H-triolein and ^3^H-triolein and ^14^C DPPC emulsions (Figures 6E-6H) were performed with incubations at 37 deg C for 30 minutes prior to reaction termination. For each of these assays, X-axis represents increasing amounts of recombinant lipase added, expressed in volume of the total reaction (up to 100 μl). The lipase assays using recombinant lipases with ^3^H-triolein labeled human VLDL was performed for four hours (Figure 6I). To prevent confusion given the differing timepoints for the data presented in Figures 6E-6H compared with the data presented in Figure 6I, we have removed reference to the assays being 30 minutes from the main text. The Methods section on “In vitro lipase assays” includes descriptions of the assay durations for Figures 6E-6H and Figure 6I respectively.

**3. Lines 248-250: This language suggests that Ser169Ala somehow affects LPL. This is clearly not the case, as it also reduces TG lipase activity in the absence of LPL (Fig. 6E). It should be stated that DN EL likely inhibits endogenous EL produced by COS7 cells.**

We agree with the Reviewer and have modified the description of the in vitro lipase activity assays to include description of the p.Ser169Ala variant in EL as a catalytically inactive variant that also inhibits any endogenous murine EL from COS7 cells and that the reduction of TG lipase activity conferred by this variant was observed both in the absence and presence of LPL co-incubation. Our revised discussion of these data is as follows (Page 11, Lines 234-252):

*While EL has been shown to be predominantly a phospholipase [1], it does also demonstrate some TG lipase activity in vitro. Our above data suggested that loss of EL activity delays lipolysis of long-chain PUFAs in both TGs and PCs. We sought to better understand the relative contributions of EL phospholipase and triglyceride lipase activities to TRL lipolysis in concert with the predominant TG lipase LPL. We generated dual ^3^H-triolein TG and ^14^C-DPPC PC labeled lipid emulsions and compared TG and phospholipase activities of the two enzymes. As previously demonstrated [1], LPL demonstrated substantially greater TG lipase activity against TRL-like emulsions than WT EL, which demonstrated a modest but dose-dependent contribution to TG lipolysis (****Figure 6E****). Likewise, WT EL demonstrated a significant dose-dependent phospholipase activity on TRL-like emulsions compared to LPL (F****igure 6F****). The combination of WT EL with LPL substantially increased PC lipolysis on TRL-like emulsions relative to that of LPL alone (****Figure 6G****) and also increased TG lipolysis above that of LPL alone (****Figure 6H****). A catalytically inactive dominant negative variant p.Ser169Ala that we previously showed to reduce WT murine EL secretion from COS7 cells in vitro and delay LDL-PC catabolism in vivo when overexpressed [2] was also tested (****Figures 6E-6H****). This variant demonstrated markedly impaired phospholipase activity on TRL-like emulsions compared with WT EL when added alone (****Figure 6F****) or in combination with LPL (****Figure 6G****). EL p.Ser169Ala also impaired TG lipolysis when incubated alone (****Figure 6E****) or in the presence of LPL (****Figure 6H****) compared with WT EL which promoted TG lipolysis in both settings (****Figures 6E and 6H****).*

**4. How do the authors explain that DN EL inhibits the TG-lipase activity of endogenously produced EL (Figs. 6E and 6H), but fails to reduce (6F) or even increases (6G) PL activity? These results should be explained, or at least acknowledged.**

The mechanisms behind the ability of the EL p.Ser169Ala human variant to reduce TG lipase activity in vitro in the absence and presence of LPL and the differential findings with respect to phospholipase activity are an important question raised by the Reviewer. Prior work from our group on this variant [2, 3] has shown that it is a catalytically inactive variant with respect to lipolytic activity for EL. Our 2004 study of LDL phospholipid catabolism by EL [2] suggested that in the presence of murine WT EL, human recombinant p.Ser169Ala EL could function as a dominant negative variant and inhibit murine EL expression in COS7 cells stably overexpressing murine EL. In the current experiments, COS7 cells used to generate recombinant human p.Ser169Ala EL were not co-overexpressing murine EL and thus the amount of endogenous EL being expressed was likely to be minimal. In this setting the phospholipase and TG lipase activity of p.Ser169Ala EL seen in Figures 6E-6G likely reflect minimal lipolysis in the setting of a catalytically inactive variant. This is consistent with the minimal and background level of TG and phospholipase activity seen for the p.Ser169Ala EL variant in HEK293 cells when overexpressed in one of our prior studies [3] (termed S149A in that study). The reduction in TG lipolysis seen with the p.Ser169Ala EL variant in the context of co-incubation with LPL is more dramatic and likely reflects the ability of the variant to impact TG lipolysis the background of markedly increased lipolysis due to LPL. The mechanisms of the p.Ser169Ala EL variant in inhibiting TG lipolysis in the presence of LPL remains unclear and is an important limitation of our prior and current studies. We do not expect that there is significant murine endogenous EL in the conditioning media containing recombinant human p.Ser169Ala EL. Thus we do not believe that the reason behind reduced TG lipolysis in the setting of p.Ser169Ala EL is primarily due to inhibition of endogenous murine EL (though this was not measured). Our prior work suggested that p.Ser169Ala EL could function through non-lipolytic functions such as lipoprotein bridging and potentially other functions in addition to being a catalytically inactive mutant. With regard to the contribution of EL catalytic activity to relative phospholipid and TG lipolysis of TG-rich lipoproteins when EL was alone or in the presence of LPL, our work here studying this variant still suggests that a variant in EL absent of WT catalytic lipolytic activity does not facilitate phospholipid or TG lipolysis in a manner seen by WT EL. We acknowledge the lack of clarity in the exact mechanism by which p.Ser169Ala functions to inhibit TG lipolysis when co-incubated with LPL is a limitation and have included this in our revised manuscript in the Discussion section. We believe the totality of the data studying the activities of EL and LPL together, both through the additional in vitro experiments using human VLDLs with EL, LPL or both (Figure 6I) and the in vivo experiment studying inhibition of LPL with ANGPLT4 AAV expression in the setting of EL deficiency in mice, support the overall claim that WT EL and EL lipolytic activity facilitates TG lipolysis with LPL. Our revised Discussion appears as follows (Pages 14-15, Lines 323-347):

*The mechanisms by which EL contributes to TRL metabolism may include both its phospholipase activity, which is its predominant lipase activity, as well as an underappreciated direct TG lipolysis. Here we show in our in vitro experiments and in lipidomic characterization of postprandial TRLs from Lipg^-/-^ mice that loss-of-function of EL impairs both phospholipid and TG lipolysis on TRLs. Since TRLs possess a phospholipid shell surrounding a neutral lipid TG core and our data and prior work suggests that EL possesses significantly higher phospholipase:TG lipase activity on a spectrum of lipoproteins, we hypothesize that one mechanism by which EL confers more efficient TG lipolysis along with LPL is through “unshielding” the exterior PCs through its phospholipase activity and allowing greater access by LPL to the TGs in the lipoprotein core. Our data on the p.Ser169Ala variant in EL also demonstrated that this catalytically inactive variant blunted both PC and TG lipolysis compared with WT EL, suggesting that the lipolytic activity of EL is an important contributor to TRL catabolism. This variant also demonstrated an ability to reduce overall TG lipolysis in the presence of robust lipolysis from LPL but without WT EL present. The mechanism behind this observation remains unclear from the current data. Prior work on this variant has suggested that it may have a dominant negative function in impairing endogenous murine EL secretion when co-overexpressed in COS7 cells [2]. It remains possible as previously speculated that a nonlipolytic function related to ligand binding may be impacted by this variant [3], which could explain why it may also impact TG lipolysis in the presence of LPL even when WT EL is not present for the variant to inhibit. Overall our data presented here is limited in its ability to dissect the relative contributions of phospholipase, TG lipase and any nonlipolytic activities of EL to determine which of these functions predominates in the ability of EL to promote TRL-TG lipolysis. Our work does demonstrate that EL has underappreciated enzymatic TG lipase activity on TRLs and that this likely contributes at least in part to the markedly enhanced TRL lipolysis seen in concert with LPL in vitro and in vivo*

**5. Fig. 6F: Figure shows PL activity, but the figure legend states TG-lipase.**

We thank the Reviewer for identifying this mistake and have corrected it in the revised manuscript.

**6. Fig. 6I is missing from the figure legends.**

We thank the Reviewer for identifying this omission and have corrected it in the revised manuscript.

**7. Fig. S3: These data provide critical in vivo support for one of the main contentions of the manuscript, i.e. the collaboration between EL and LPL. As such, these figures would be more appropriate in the main body of the paper.**

We agree with the Reviewer’s suggestion and have moved Figure S3 to become Figure 7 of the revised manuscript and have changed Figure 7 from the prior version of the manuscript to Figure 8.

**8. Figs. 6I and S3F: The use of one-sample T-test is inappropriate, because it assumes a known theoretical value as a comparison and ignores the variation in measured values. Thus, this test will inflate the significance of differences. In Fig. 6I, one-way ANOVA should be used to evaluate the difference of EL+LPL from the other two groups. The large increase above the sum of means (red line) provides strong support for an interaction between the two lipases, even without further statistical analysis. In Fig. S3F, the significance of interaction term (genotype x AAV) in 2-way ANOVA should be assessed.**

We thank the Reviewer for important insights in how to best present the data summarized in Figure 6I and Figure S3F (now Figure 7F). We have removed one-sample T tests as recommended and have revised the analysis to analyze Figure 6I by one-way ANOVA and Figure 7F by two-way ANOVA. We have retained the red lines to demonstrate the increase of the relative lipase activity in vitro for the combined lipase assays relative to the sum of the individual lipases (Figure 6I) and relative increase in postprandial TGs in the ANGPTL4 AAV expressing *Lipg*^-/-^ mice relative to the sums of increased TGs in the ANGPTL4 AAV expressing WT and Null AAV expressing *Lipg*^-/-^ mice (Figure 7F).

**9. Fig. S3E: The TG scale seems to be off by a factor of 10.**

We appreciate this point brought up by the Reviewer. We reviewed our TG measurements as assessed by colorimetric assay from this oral fat tolerance experiment and confirmed the TG measurements are correct as listed. Variation in the TG levels may reflect systematic differences in our assay standard used for this set of measurements, or the relatively young age of these mice (6-8 weeks old, compared with 10-12 weeks for mice used in oral fat tolerance testing described in Figure 4A or older mice after high fat feeding as seen in Figure 4C), which impacts total volume of olive oil gavaged in these mice (10 µL/g) and duration of clearance of chylomicrons. We have acknowledged the difference in the weights of the mice for this experiment which was done as part of our initial resubmission in our Materials and Methods section. We show relative differences in postprandial TGs as seen in Figure 7F.

**References**

1.	McCoy MG, Sun GS, Marchadier D, Maugeais C, Glick JM, Rader DJ. Characterization of the lipolytic activity of endothelial lipase. Journal of lipid research. 2002;43(6):921-9. Epub 2002/05/29. PubMed PMID: 12032167.

2.	Broedl UC, Maugeais C, Millar JS, Jin W, Moore RE, Fuki IV, et al. Endothelial lipase promotes the catabolism of ApoB-containing lipoproteins. Circulation research. 2004;94(12):1554-61. Epub 2004/05/01. doi: 10.1161/01.res.0000130657.00222.39. PubMed PMID: 15117821.

3.	Broedl UC, Maugeais C, Marchadier D, Glick JM, Rader DJ. Effects of nonlipolytic ligand function of endothelial lipase on high density lipoprotein metabolism in vivo. The Journal of biological chemistry. 2003;278(42):40688-93. Epub 2003/08/12. doi: 10.1074/jbc.M304367200. PubMed PMID: 12909635.
